# Spontaneous Massive Adrenal Hemorrhage: A Management Dilemma

**DOI:** 10.1089/cren.2015.0003

**Published:** 2015-11-01

**Authors:** Praveen Pushkar, Anshuman Agarwal

**Affiliations:** Department of Urology, Indraprastha Apollo Hospital, New Delhi, India.

## Abstract

Adrenal hemorrhage (AH) is a rare but life-threatening condition. Small focal hemorrhage may present subclinically, but massive hemorrhage may lead to rapid cardiovascular collapse and ultimately death if not diagnosed appropriately and treated quickly. Most cases reported in the literature have been treated conservatively. In an event of increasing hemorrhage during conservative management, it may be tricky to intervene surgically because of the hematoma around the gland. Here we describe a case where we managed a large spontaneous AH by a combination of angioembolization and laparoscopic adrenalectomy.

## Introduction and Background

Adrenal hemorrhage (AH) is a rare but life-threatening condition, especially when it occurs bilaterally. Etiology includes both traumatic and nontraumatic events. Clinical manifestation depends on the degree and rate of hemorrhage. Small focal hemorrhage may present subclinically, but massive hemorrhage may lead to rapid cardiovascular collapse and ultimately death if not diagnosed appropriately and treated quickly. Most cases reported in the literature have been treated conservatively. In an event of increasing hemorrhage during conservative management, it may be tricky to intervene surgically because of the hematoma around the gland. Here we describe a case where we managed a large spontaneous AH by a combination of angioembolization and laparoscopic adrenalectomy. To the best of our knowledge, it has not been reported in the literature yet.

## Presentation of Case

**FIG. 1.**
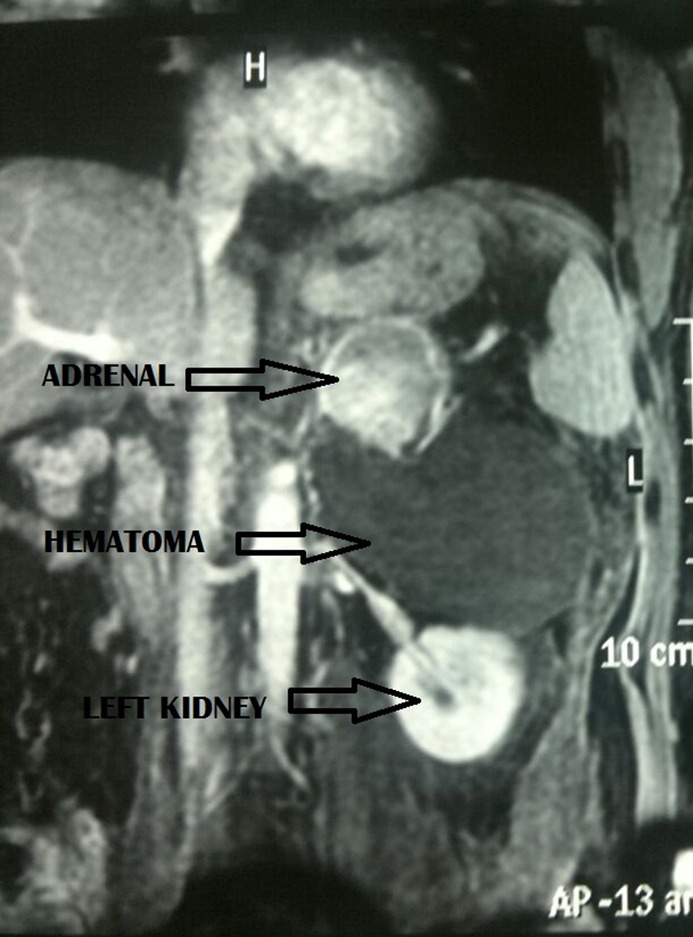
MRI showing left suprarenal mass with large hemorrhagic component, suggestive of spontaneous hemorrhage in an adrenal mass, likely representing pheochromocytoma.

**FIG. 2.**
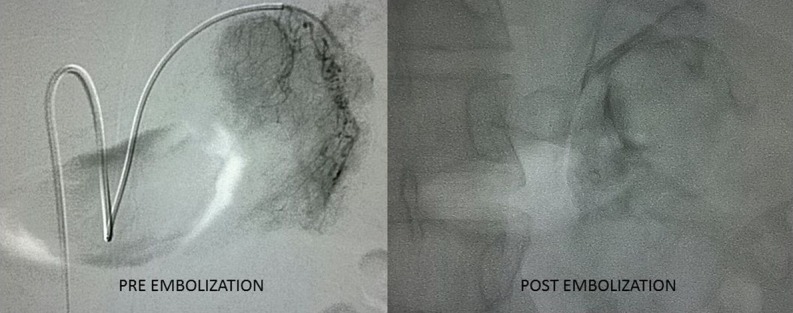
Angiography revealed a highly vascular adrenal tumor. Two arteries were identified, one arising from aorta supplying superior part of gland and other arising from accessory left renal artery supplying the inferior part (pre-embolization). Both the arteries were embolized (postembolization).

A 26-year-old, nonhypertensive young lad presented to us in emergency with left-sided acute abdominal pain of 1 day duration. Pain was sudden in onset, severe, continuous, and nonradiating. On initial examination, he was hemodynamically stable with blood pressure of 140/70 mm Hg and heart rate of 110 beats per minute. On abdominal examination, he had a tender vague lump in the left upper quadrant of abdomen. Bowel sounds were normal. First, ultrasound (USG) abdomen and, later, contrast-enhanced CT scan abdomen were performed, which revealed hemorrhagic collection of 9 × 8.2 × 8.2 cm near the left suprarenal region with 4 × 4.5 × 4.2 cm adrenal mass. To evaluate the adrenal mass, MRI was performed, which showed the left suprarenal mass with large hemorrhagic component, suggestive of spontaneous hemorrhage in an adrenal mass (Fig. 1). Based on imaging finding, a working diagnosis of pheochromocytoma was made and he was started on phenoxybenzamine 10 mg twice a day to prepare him for surgery. His urinary catecholamines were sent before starting phenoxybenzamine. He was kept in intensive care unit for close monitoring. There was one blood pressure recording of >160 mm Hg (systolic). He complained of resurgence in pain, so a bed-side USG of the abdomen was performed, which revealed an increase in collection by 500 mL resulting in a drop of Hb by 2 gm/dL. He was transfused 2 units of packed red blood cells. Immediately, angioembolization was performed using Gelfoam (Fig. 2). No blood pressure variation was recorded during the procedure. Thirty-six hours later, he was taken up for laparoscopic exploration with adrenalectomy. Laparoscopy revealed a large collection of clotted blood in the retroperitoneum pushing the kidney inferiorly. Fortunately, there was no active hemorrhage. The ruptured adrenal gland was isolated after evacuation of the hematoma and removed after securing the pedicles. There was no blood pressure fluctuation during the surgery. Postoperative period remained uneventful. Results of urinary catecholamine levels came after the surgery and were normal. He was discharged on postoperative day 5. Histopathologic examination revealed adrenocortical adenoma based on the Weiss criteria. Follow-up performed after 6 and 12 months revealed no abnormality in positron emission tomography CT scan.

## Discussion and Literature Review

AH may result from acute illness/stress, anticoagulation, coagulopathy, underlying tumor-like angiomyolipoma, trauma, or idiopathic disease. Most of the reported cases of AH in the literature are seen in pregnancy. These are usually intra-AHs. In young adults, idiopathic spontaneous AH is extremely rare and has never been reported. The presenting symptoms usually are hemorrhagic shock, flank pain, and fever.

AH has been reported in 0.3% to 1.8% of undetected cases in autopsy studies.^1^ Tumors known to cause spontaneous bleed are pheochromocytoma, myelolipoma, metastasis, carcinoma, and rarely adenoma. There are ∼50 reported cases of spontaneous rupture of adrenal pheochromocytoma causing haemorrhage^2^ in the literature, but there is no reported case of adrenal adenoma causing massive bleeding.

Once adrenal pathology is suspected in a patient with retroperitoneal hemorrhage, he should be managed in an intensive care unit with close hemodynamic monitoring. Serial hematocrit and USG monitoring of size of hematoma has to be performed. Pheochromocytoma should always be kept in mind while dealing with such a case. MRI is the imaging modality of choice for diagnosis of nontraumatic AH.

In patients with active bleeding, angiographic embolization is a valuable tool to achieve hemostasis. If complete hemostasis is achieved, patient is asymptomatic, and hemodynamically stable, then immediate surgical exploration should be avoided. But if patient deteriorates, surgical exploration may have to be performed. All preoperative principles performed in pheochromocytoma should be followed.

This is the first case report of such a massive spontaneous AH in adrenal adenoma in a young adult, which was effectively managed laparoscopically.

## Conclusion

A high index of suspicion is required to make a timely diagnosis of AH. Although acute surgical removal of an adrenal tumor within a large hematoma should be avoided, as proper oncologic resection may not be possible, it sometimes has to be performed in a symptomatic hemodynamically deteriorating patient.

## Abbreviations Used

AH: adrenal hemorrhage
CT: computed tomography
MRI: magnetic resonance imaging
USG: ultrasound
